# Crosstalk between exosomes and tumor-associated macrophages in hepatocellular carcinoma: implication for cancer progression and therapy

**DOI:** 10.3389/fimmu.2025.1512480

**Published:** 2025-04-08

**Authors:** Ying Xu, Linyue Xu, Qiuyan Chen, Can Zou, Ju Huang, Limei Zhang

**Affiliations:** ^1^ Department of Anesthesiology Operating Room, Hospital of Chengdu University of Traditional Chinese Medicine, Chengdu, Sichuan, China; ^2^ School of Sports Medicine and Health, Chengdu Sport University, Chengdu, China; ^3^ Department of Respiratory Medicine, Hospital of Chengdu University of Traditional Chinese Medicine, Chengdu, Sichuan, China

**Keywords:** exosome, tumor-associated macrophage, tumor microenvironment, hepatocellular carcinoma, immunotherapy

## Abstract

Hepatocellular carcinoma (HCC), the most prevalent type of primary liver cancer, represents a significant cause of cancer-related mortality. While our understanding of its pathogenesis is comparatively comprehensive, the influence of the tumor microenvironment (TME) on its progression warrants additional investigation. Tumor-associated macrophages (TAMs) have significant impacts on cancer cell proliferation, migration, invasion, and immune response, facilitating a complex interaction within the TME. Exosomes, which measure between 30 and 150 nanometers in size, are categorized into small extracellular vesicles, secreted by a wide range of eukaryotic cells. They can transfer biological molecules including proteins, non-coding RNAs, and lipids, which mediates the intercellular communication within the TME. Emerging evidence has revealed that exosomes regulate macrophage polarization, thus impacting cancer progression and immune responses within the TME of HCC. Moreover, TAM-derived exosomes also play crucial roles in malignant transformation, which hold immense potential for cancer therapy. In this review, we elaborate on the crosstalk between exosomes and TAMs within TME during HCC development. Moreover, we delve into the feasible treatment approaches for exosomes in cancer therapy and emphasize the limitations and challenges for the translation of exosomes derived from TAMs into clinical courses for cancer therapy, which may provide new perspectives on further ameliorations of therapeutic regimes based on exosomes to advance their clinical applications.

## 1 Introduction

According to the 2020 GLOBOCAN database, liver cancer ranks as the seventh most prevalent cancer with 905,677 new cases globally, accounting for approximately 4.7% of all cancer types (1). This presents a significant burden on global healthcare systems. Liver cancer primarily includes hepatocellular carcinoma (HCC), intrahepatic cholangiocarcinoma, and other rare forms, with HCC accounting for approximately 85% of all liver cancer cases (2). Treatment regimes for advanced HCC include systemic chemotherapy, targeted therapy, arterial chemoembolization, and emerging strategies like immunotherapy (3, 4). However, drug resistance and limited therapeutic responses are significant challenges for improving patients’ survival (5). Therefore, there is an urgent need for new scientific and technological approaches for integrated diagnosis and treatment of HCC in clinical practice.

The tumor microenvironment (TME) is a fundamental component of the tumor ecosystem, serving as the site where tumor cells interact with both other tumor cells and host cells (6). By orchestrating changes in the TME—including stromal cells, immune cells, and immune regulatory molecules—a pro-tumor TME is created, leading to tumor growth and hinder the effectiveness of anti-cancer therapies (7). Tumor-associated macrophages (TAMs) are among the most prevalent immune cell types within the TME of HCC, which secrete a diverse range of biological factors, including inflammatory molecules, chemokines, and exosomes, thereby impacting tumor cell proliferation, migration, invasion and immune response (8).

Exosomes, which measure between 30 and 150 nanometers in size, are categorized into small extracellular vesicles, secreted by a wide range of eukaryotic cells (9). While exosomes were first identified in the late 1980s, they were initially regarded merely as waste disposal mechanisms (10). However, advances in biotechnology have led to the recognition that exosomes are widely distributed in human body fluids and play an essential role in intercellular communication (11). Exosomes, arising from the endosomal pathway through the creation of late endosomes or multivesicular bodies, encapsulate a diverse array of molecules specific to their parent cells (12). These molecules can be transported over considerable distances while being shielded within a lipid bilayer-enclosed structure (13). Recently, research on the role of exosomes in cancer progression has received tremendous attention due to their ubiquitous presence and easy accessibility, they offer considerable potential for the advancement of precision medicine (14). Exosomes derived from TAMs account for a significant proportion of the blood, offering new clinical biomarkers for minimally invasive liquid biopsies in HCC patients (15). TAMs-derived exosomes transport non-coding RNAs (ncRNAs), proteins, and lipids that modulate malignant cell proliferation, metastasis, metabolic reprogramming, and immune response in the setting of HCC models (16, 17). Therefore, TAMs-derived exosomes hold immense potential for the systematic therapy of HCC. In this review, we emphasize the crosstalk between exosomes and TAMs, concentrating on the role of exosomes in macrophage polarization and their molecular functions on the cell proliferation, metastasis, and immune responses in the TME of HCC. Furthermore, novel therapeutic strategies based on exosomes and challenges faced in the clinical applications are also proposed, aiming to provide novel biomarkers and therapeutic targets in the field of HCC.

## 2 The role of macrophages in HCC

Macrophages, essential elements of the innate immune system, are widely present in the bloodstream and across multiple tissues in the body (18). Macrophages display remarkable plasticity, allowing them to adjust to a wide range of tissue microenvironments and carry out multiple functions including presenting antigens, clearance of target cells and pathogens, and immune regulation (19). They are also capable of swiftly sensing and integrating various signals from their microenvironments, thereby contributing to the maintenance of homeostasis (20). Liver macrophages can be categorized into two types based on their origin: Kupffer cells (KCs), which are tissue-resident macrophages, and macrophages derived from monocytes (21, 22). However, in the process of hepatocarcinogenesis, pro-tumorigenic molecules stimulate and activate them for phenotypic shift, resulting in the transformation into TAMs (23).

Macrophages are classified into two polarized states, M1 and M2, depending on their activation status (24). M1 macrophages are primarily characterized by their pro-inflammatory effects and the production of substantial quantities of pro-inflammatory mediators (25). Their classical activation occurs in response to various stimuli, such as 1) lipopolysaccharides, 2) interferon-γ (IFN-γ), 3) tumor necrosis factor (TNF), 4) granulocyte-macrophage colony-stimulating factor (GM-CSF), and 5) Toll-like receptor (TLR) ligands (26–28). Upon activation, M1 macrophages secrete interleukins, chemokines, and TNF-α, all of which contribute to pro-inflammatory responses (29, 30). Additionally, they are capable of exerting cytotoxic effects by producing nitric oxide (NO) and reactive oxygen species (ROS) through the enzymes NOS2 or iNOS (31, 32). M1 macrophages that express high levels of MHC-II are essential for regulating Th-1-type immune responses (33). In a subsequent phase, M1 macrophages are influenced by Th2 cytokines such as interleukin 4 (IL-4) and IL-13 to polarize at tumor sites, leading to their transformation into M2-type macrophages (34, 35). These M2 macrophages produce anti-inflammatory cytokines such as IL-10 and transforming growth factor-β (TGF-β) (36, 37). M2-type macrophages are marked by elevated expression levels of CD206, CD163, and TGFβR, primarily functioning to inhibit inflammatory responses, which can facilitate tumor growth and metastasis (38, 39). Furthermore, M2 macrophages impact various cell types within the TME, including cancer-associated fibroblasts (CAFs), endothelial cells (ECs), dendritic cells (DCs), natural killer (NK) cells, and myeloid-derived suppressor cells (MDSCs) (40, 41). Notably, the M1-M2 polarization is a highly dynamic and reversible process. Within the TME, macrophages that are designated as TAMs, predominantly of the M2 subtypes, play a pivotal role in tumor progression (40, 42). TAMs secrete a diverse array of cytokines and inflammatory factors, enhancing interactions with other cell types in the TME, and thereby promoting tumor metastasis, angiogenesis, and mechanisms of immune evasion (43, 44).

Classically activated (M1 type) macrophages frequently display anti-tumor characteristics. In the context of HCC, Sirtuin 1 (SIRT1) was capable of enhancing the infiltration of M1-like macrophages and suppressing HCC metastasis by NF-κB pathway (45). Interleukin 12 (IL-12) facilitated the conversion of monocytes into an M1-like phenotype through the inhibition of the signal transducer and activator of transcription 3 (STAT3) pathway. This transformation markedly downregulated pro-tumoral molecules, including TGF-β, vascular endothelial growth factor (VEGF)-A, and MMP-9, resulting in the suppression of tumor cell growth and metastasis, as well as a notable reduction in xenograft tumor growth *in vivo*(46).

**Figure 1:**
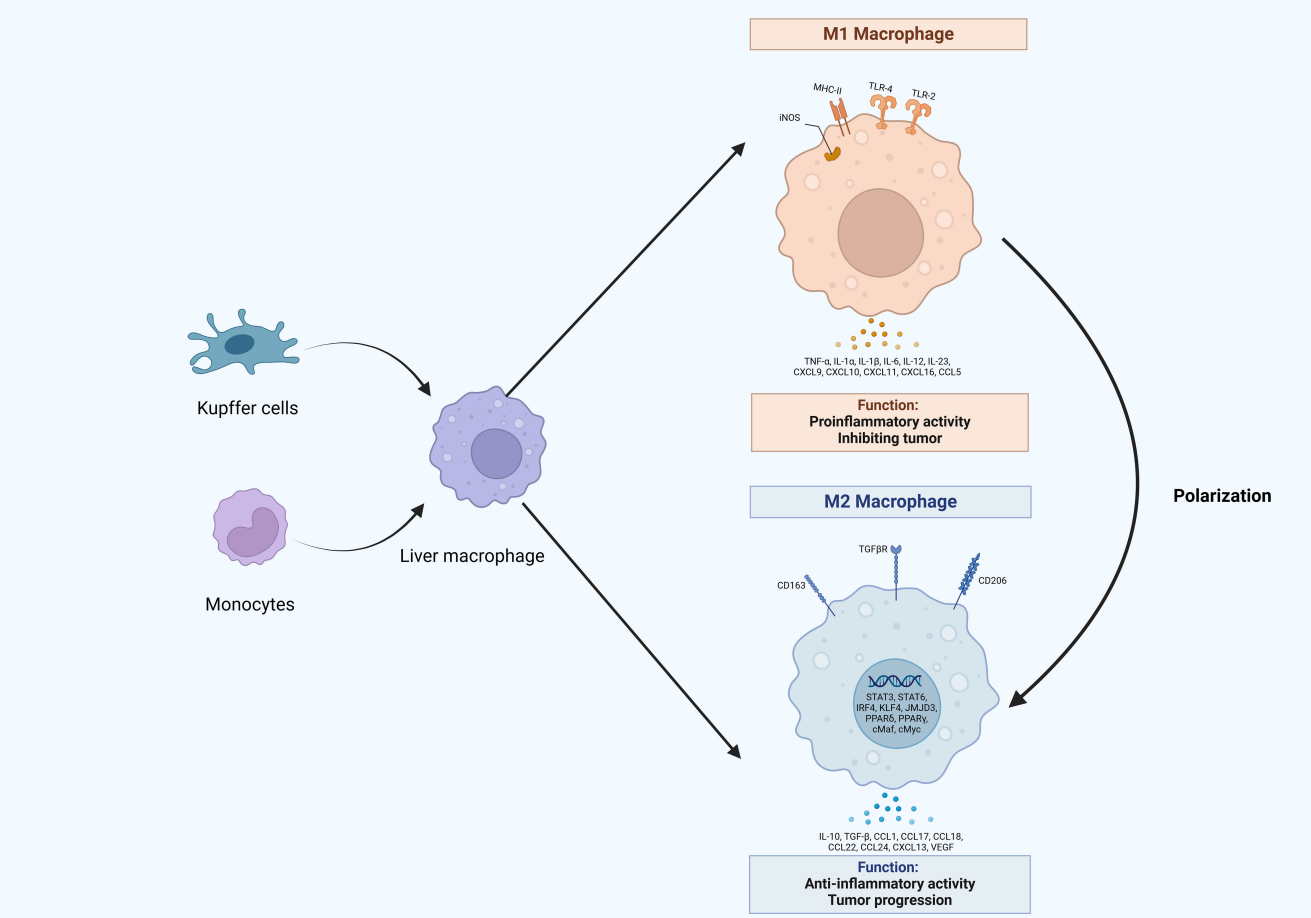
The origin and polarization of macrophages in hepatocellular carcinoma. Liver macrophages originate from Kupffer cells and monocytes, which infiltrate tumors and differentiate into tumor-associated macrophages (TAMs). TAMs would undergo different activation processes that differentiate into M1 or M2 macrophages, which release various molecules that display different functions.

Alternatively activated macrophages (M2) could secrete the cytokine C-C Motif Chemokine Ligand 22 (CCL22), which enhanced tumor cell metastasis through the activation of the Smad pathway, as well as the upregulation of Snail (47). IL-25 induced M2 macrophages activation and promoted the secretion of C-X-C motif Chemokine Ligand 10 (CXCL10), leading to the facilitated HCC progression (48). Transmembrane protein 147 (TMEM147) interacted with 7-dehydrocholesterol reductase (DHCR7) and enhanced its expression by promoting the STAT2 pathway, thereby conferring ferroptosis resistance and facilitating macrophage polarization into the M2-like phenotype to promote tumor growth and invasion in HCC (49) (**Figure 1**).

In recent years, single-cell RNA sequencing (scRNA-seq) has been widely used to study tumor heterogeneity. Emerging studies have revealed that TAMs exhibited a combination of both canonical M1-like genes and M2-like genes (50, 51). These findings challenged the traditional polarization theory of macrophages, which posited that M1 and M2 polarization states exist at opposite ends of a spectrum. A novel framework has been proposed for categorizing macrophages that incorporates functional characteristics. For example, Yang and colleagues revealed that CK19-positive HCC possessed an inhibitory TAM niche and identifies, for the first time, a significant enrichment of specific SPP1-positive TAMs in CK19-positive HCC (52). Although SPP1^+^ TAMs were identified as the dominant macrophage type within the immune barrier of HCC, DAB2^+^ TAMs exhibit a higher infiltration in HCC. DAB2^+^ TAMs primarily originate from hepatic Kupffer-like cells, whereas SPP1^+^ TAMs are more likely derived from monocyte-like macrophages, indicating potential functional differences between these populations. While both may promote extracellular matrix remodeling through the TGF-β signaling pathway, PDGFB and ADM have been identified as specific ligands for DAB2^+^ TAMs and SPP1^+^ TAMs, respectively, exerting distinct exclusive functions (53). This thorough understanding of macrophage classification is paving the way for a new era of therapeutic targeting, resulting in enhanced efficacy of treatment strategies.

## 3 Biological characteristics and properties of exosomes

**Figure 2:**
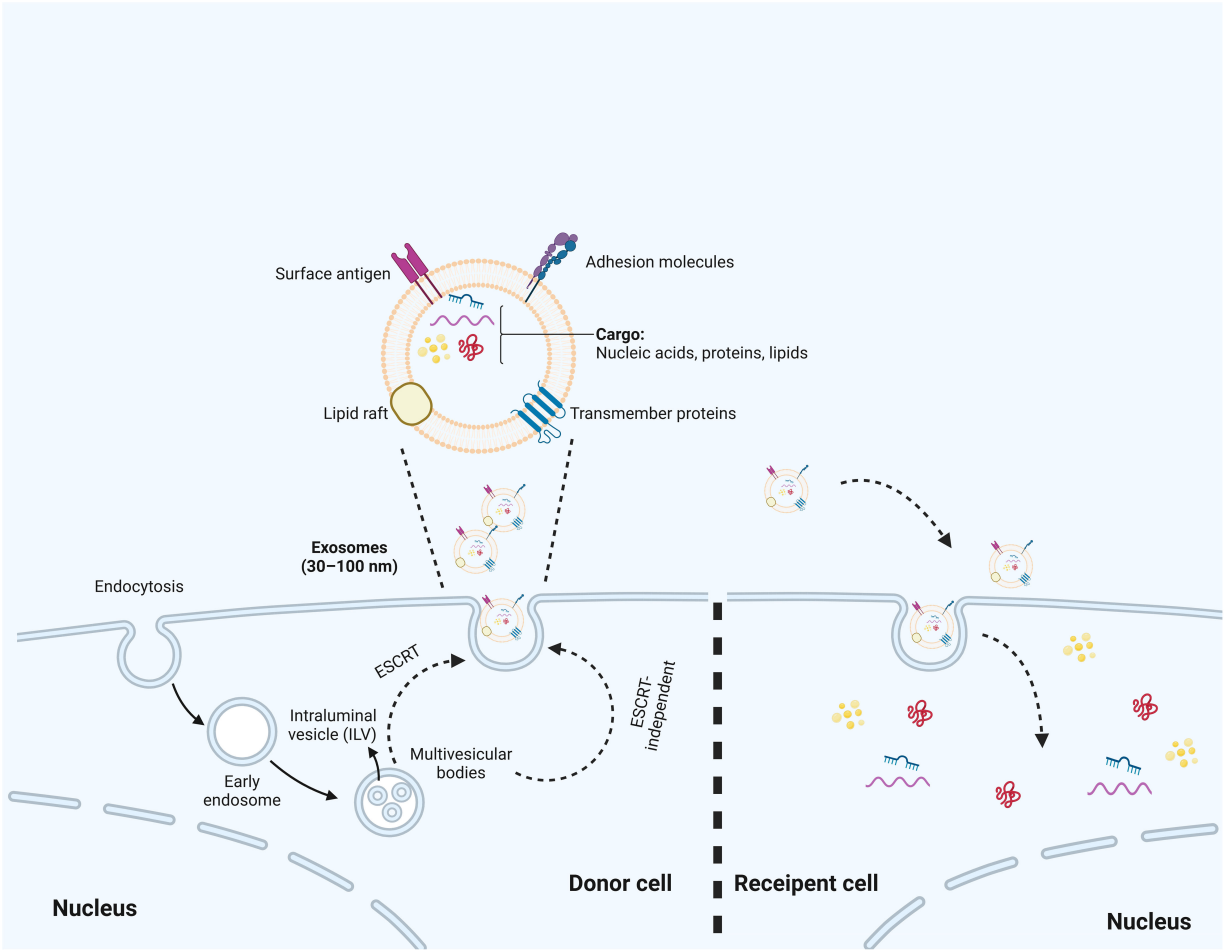
The biosynthesis process of exosomes. The biosynthesis process begins with the inward budding of the cell membrane, which leads to the formation of early endosomes. Subsequently, multivesicular bodies (MVBs) are created through additional inward budding of these endosomes, during which various miRNAs, proteins, and other selected substances are incorporated. Ultimately, MVBs can either fuse with the cell membrane, resulting in the inclusion of extracellular DNA, or merge with lysosomes, leading to the degradation of the biological information contained within the MVBs. Additionally, the production of exosomes are associated with ESCRT or ESCRT independent pathways.

Exosomes are extracellular vesicles characterized by a double-membrane structure, formed through the outward budding of the plasma membrane, and can be naturally found in blood, cerebrospinal fluid, and urine (54–56). The process of exosome biogenesis initiates with the inward invagination of the plasma membrane, leading to the formation of endosomes, which are known as multivesicular bodies (MVBs) (57). Within these endosomes, the membranes undergo further invagination to create smaller vesicles, typically ranging from 30 to 150 nm in size, referred to as intraluminal vesicles (ILVs) (58). Proteins, lipids, and nucleic acids are selectively sorted and encapsulated in ILVs (59). This process is driven by the endosomal sorting complex required for transport (ESCRT) (60). ESCRT consists of four complexes: ESCRT-0, -I, -II, and -III, which regulate the formation of ILVs and sort cargoes into specific microdomains of the limiting membrane of MVBs (61, 62). The ESCRT machinery functions sequentially. Phosphatidylinositol 3-phosphate activates ESCRT-0, which comprises hepatocyte growth factor-regulated tyrosine kinase substrate (HRS), a protein that identifies ubiquitinated proteins and associates with STAM, another member of the ESCRT-0 complex. HRS is capable of bringing tumor susceptibility gene 101 (TSG101) into the ESCRT-I complex (63). ESCRT-I then recruits ESCRT-II, activating ESCRT-III to cleave the endosomal membrane (64). Finally, ESCRT-III and the AAA ATPase Vps4, facilitate the de-ubiquitination of cargoes and the detachment of ESCRT-III from the endosomal membrane (65). Moreover, ESCRT-independent mechanisms also play an essential role in exosome biogenesis, which is facilitated by sphingomyelins (66). It promotes the formation of lipid raft microdomains, which contribute to the production of ILVs (67). Subsequently, the MVBs that contain these ILVs subsequently fuse with the plasma membrane or undergo degradation by lysosomes and autophagosomes, resulting in the release of the ILVs that encapsulate specific cargo, collectively identified as exosomes (57) (**Figure 2**).

Exosomes contain non-specific proteins as well as tissue-specific proteins, such as β-catenin, intercellular adhesion molecule 1 (ICAM-1) on B cells, and cytoskeletal proteins (68, 69). The lipid composition of these exosome membranes include phosphatidylcholine, phosphatidylethanolamine, and sphingomyelin (70). The tetraspanins CD9, CD81, and CD63 are key components of exosomes; however, their roles in influencing exosome composition remain insufficiently explored. In the MCF7 breast cancer cell line, CD63 was predominantly localized within the cell as anticipated. In contrast, CD9 and CD81 showed significant colocalization at the plasma membrane, displaying varying ratios at different locations, which may account for the higher prevalence of CD81 in exosomes. Notably, the absence of these tetraspanins had a negligible effect on the protein composition of exosomes as assessed through quantitative mass spectrometry (71). Additionally, exosomes harbor various types of RNA, including messenger RNA and ncRNAs (72). The loading of ncRNAs into exosomes is a highly regulated and selective process that encompasses several vital steps. The initial step involves specific RNA-binding proteins (RBPs) that recognize and bind to ncRNAs intended for exosomal packaging (73). Proteins such as heterogeneous nuclear ribonucleoprotein and Ago2 identify unique motifs or secondary structures within ncRNAs, including miRNAs, thereby facilitating their selective incorporation into exosomes (10, 74).The selective nature of the packaging process guarantees that only specific ncRNAs are loaded into exosomes. This selectivity is achieved through precise regulation of ncRNA binding by RBPs and their subsequent incorporation into the developing exosomes (75, 76). Among these RNAs, microRNA (miRNA) is the most abundant RNA type found in exosomes, which can influence the transcriptome of recipient cells (77). After their release from donor cells into the extracellular environment, exosomes can modulate recipient cell functions through direct ligand-receptor interactions, fusion with the plasma membrane, or endocytosis (9, 78). Nevertheless, the underlying mechanisms of exosome uptake and their intercellular trafficking remain to be fully understood.

For an extended period, exosomes are primarily viewed as a mechanism for the transport of cellular waste. However, recent advancements in mass spectrometry and next-generation sequencing have substantially improved our understanding of exosomal contents (79, 80). In recent years, a variety of methods for exosome isolation and purification have been developed (81). These notable advancements in methodologies and experimental approaches have significantly enhanced our comprehension of the biogenesis and functions of exosomes (80). Exosomes serve as vital carriers for signaling molecules, establishing a novel system for intercellular information transfer, and playing a crucial role in various physiological and pathological processes such as cell proliferation, differentiation, migration, and communication between cells (78). For example, Qiao et al. discovered that M2-TAMs in esophageal cancer could secret exosomal LINC01592, which coordinated with E2F Transcription Factor 6 (E2F6), leading to increased degradation of major histocompatibility complex (MHC) class I on the surface of cancer cells (82). Consequently, this enabled cancer cells to evade immune attacks from cytotoxic T lymphocytes, thereby promoting tumor growth *in vivo*(82). Furthermore, circTMCO3 was delivered to ovarian cancer cells via exosomes secreted by TAMs. Exosomal circTMCO3 functioned as the molecular sponge for miR-515-5p, therefore upregulating ITGA8, which significantly promoted ovarian malignancy in mouse models (83). These findings suggested that a therapeutic approach targeting this axis could have great potential for treating malignant disease.

Exosomes can be isolated from body fluids and are capable of being stored at -80°C for extended periods, exhibiting a relatively long lifespan (84). This makes them promising candidates as diagnostic markers and prognostic indicators in bodily fluid analysis. Additionally, exosomes are naturally occurring, demonstrating good biocompatibility and low immunogenicity, making them suitable as endogenous carriers. Based on these functional characteristics, exosomes are expected to become important tools for cancer immunotherapy, and precision medicine.

In HCC, exosomes may serve as novel, noninvasive biomarkers for cancer detection. In comparison to conventional indicators, exosomes are stable in blood and other bodily fluids, providing the benefits of minimal invasiveness and easy sample collection (85). Arbelaiz et al. reported that the exosomal galectin-3 binding protein (G3BP) was significantly elevated in HCC patients when compared to healthy controls and cholangiocarcinoma patients, showcasing an area under the curve (AUC) of 0.904 and 0.894, respectively (86). Numerous exosomal proteins also demonstrate potential as prognostic indicators, enabling predictions of survival and recurrence rates in HCC patients. S100A4 is a critical component found in HCC exosomes that promoted tumor metastasis by activating STAT3 and inducing osteopontin production (87). Researchers have examined the levels of exosomal S100A4 in relation to survival and recurrence, discovering that the combination of exosomal S100A4 and osteopontin levels provides a better predictive performance than AFP alone (87). Additionally, adenylyl cyclase-associated protein 1 (CAP1) showed a correlation with HCC metastasis and was significantly valued in exosomes. Consequently, exosomal CAP1 is proposed as a potential diagnostic marker for HCC and merits further investigation (88). Researchers must remain committed to advancing this field to uncover the clinical applications of exosomal biomarkers for HCC.

## 4 Interaction of tumor cell-derived exosomes and macrophages in the microenvironment of HCC

**Figure 3:**
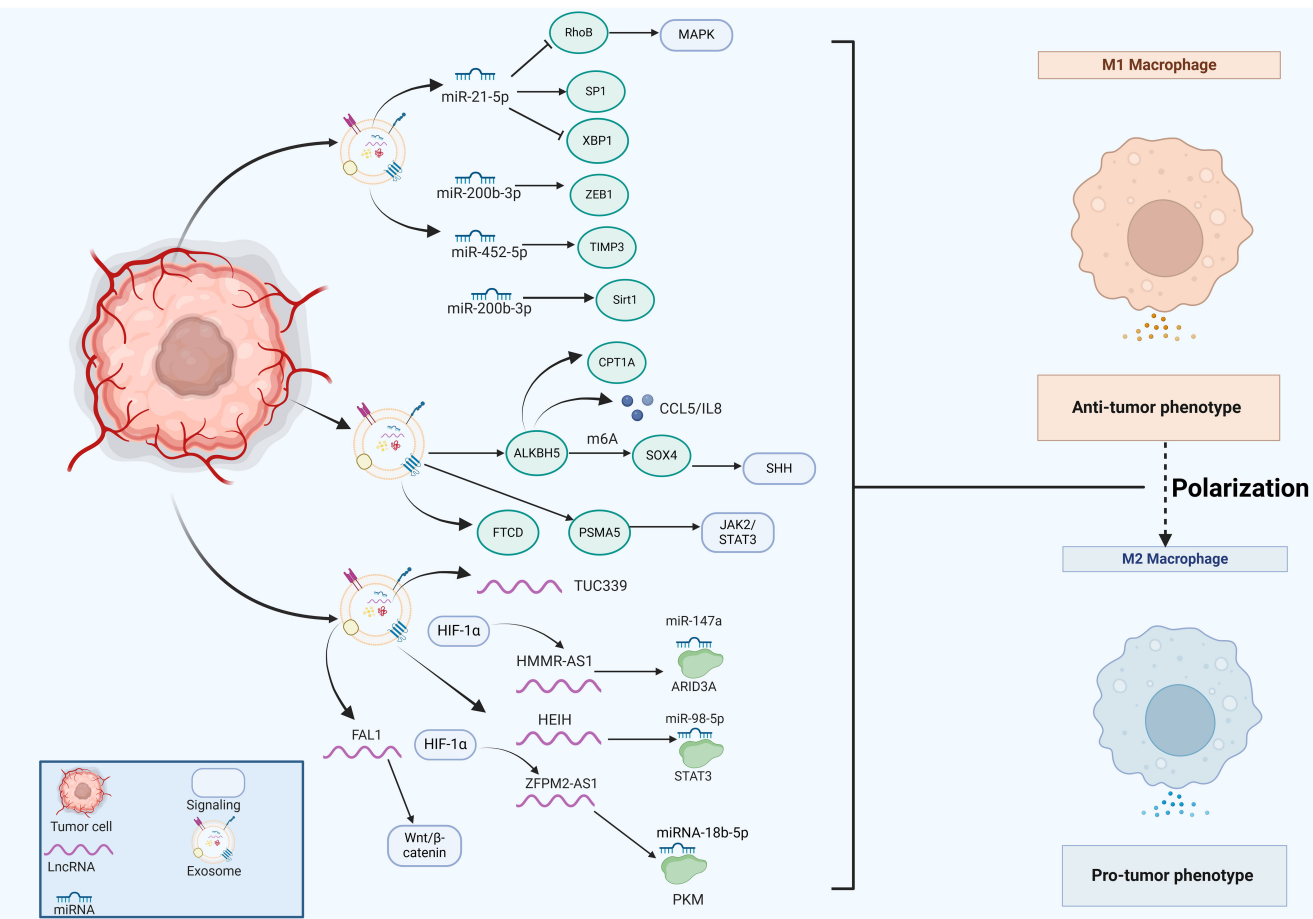
The emerging role of tumor cell-derived exosomes in HCC progression by regulating macrophage polarization. Tumor cells secret exosomes that contain miRNAs (miR-21-5p, miR-200b-3p, miR-452-5p), lncRNAs (TUC339, HMMR-AS1, HEIH, ZFPM2-AS1, FAL1) and various proteins (ALKBH5, FTCD, PSMA5), which significantly impact macrophage polarization, thus regulating HCC progression.

Research has increasingly shown that not only exosomes derived from tumor cells influence the immune cells within TME, but exosomes originating from immune cells can also impact tumor cells or other immune cells, primarily targeting TAMs. Another important role of exosomes is their ability to regulate macrophage polarization within the microenvironment of HCC (**Table 1**; **Figure 3**).

**Table 1:** The crosstalk between exosome and tumor-associated macrophages in impacting hepatocellular carcinoma progression.

Exosomal cargo	Donor cell	Mechanism	Effect	Reference
miR-21-5p	Tumor cell	Inhibited RhoB expression and suppressed MAPK pathway	Promoted M2 macrophage polarization and cancer progression	(89)
miR-21-5p	Tumor cell	Modulated SP1/XBP1 axis	Enhanced M2 macrophage polarization and promoted cancer progression	(90)
miR-452-5p	Tumor cell	Targeted TIMP3	Enhanced M2 macrophage polarization and promoted cancer progression	(91)
miR-4669	Tumor cell	Upregulated sirtuin 1 expression	Enhanced M2 macrophage polarization and promoted acquired resistance to sorafenib.	(92)
miR-200b-3p	Tumor cell	Downregulated ZEB1 expression, promoted IL-4 production, and activated JAK/STAT pathway	Enhanced M2 macrophage polarization and augmented tumor growth and metastasis.	(93)
circUPF2	Tumor cell	Facilitated the formation of the IGF2BP2-SLC7A11 ternary complex	Increased sorafenib resistance and inhibited ferroptosis	(95)
LncRNA TUC339	Tumor cell	Regulated cytokine-cytokine receptor binding	Enhanced M2 macrophage polarization	(96)
LncRNA HMMR-AS1	Tumor cell	Targeted miR-147a/ARID3A axis	Enhanced M2 macrophage polarization and promoted tumor cell proliferation	(97)
LncRNA HEIH	Tumor cell	Targeted the miR-98-5p/STAT3 axis	Enhanced M2 macrophage polarization and promoted cancer progression	(98)
FAL1	Tumor cell	Activated the Wnt/β-catenin pathway	Induced M2 macrophage polarization and promoted cancer progression	(99)
SLC16A1-AS1	Tumor cell	Enhanced the stability of SLC16A1 mRNA in macrophages	Induced M2 macrophage polarization and promoted cancer progression	(101)
miR4458HG	Tumor cell	Interacted with IGF2BP2, enhancing the stability of HK2 and GLUT1,	Induced M2 macrophage polarization and promoted cancer progression	(102)
ZFPM2-AS1	Tumor cell	Regulated glycolysis by targeting the miRNA-18b-5p/PKM axis under hypoxia conditions	Augmented the abilities and stemness of HCC cells by contributing to M2 macrophage polarization	(103)
ALKBH5	Tumor cell	Upregulated SOX4 expression, facilitated SHH pathway, promoted CCL5 secretion, upregulated IL-8 and CPT1A	Enhanced M2 macrophage polarization and promoted cancer progression	(104–107)
FTCD	Tumor cell	Directly promoted M1 macrophage polarization	Inhibited cancer progression	(109)
PSMA5	Tumor cell	Promoting JAK2/STAT3 pathway	Enhanced M2 macrophage polarization and promoted cancer progression	(110)
LncMMPA	TAM	Sponged miR-548 s and upregulated ALDH1A3 expression	Enhanced M2 macrophage polarization and promoted tumor glycolysis and growth	(17)
hsa_circ_0004658	TAM	Targeted miR-499b-5p/JAM3 axis	Inhibited cancer progression	(112)
miR-27a-3p	TAM	Inhibited TXNIP expression	Enhanced tumorigenicity, stemness, and drug resistance of cancer cells	(113)
MNDA	TAM	Promoted exosomal proteins secretion including MMP14, and TIMP	Enhanced cancer progression	(117)
miR-660-5p	TAM	Decreased expression of KLF3	Enhanced cancer progression	(118)
miR-6876-5p	TAM	Promoted EMT by targeting PTEN and activated the AKT signaling pathway.	Enhanced tumor metastasis	(119)
miR-375	TAM	miR-375 was found to be upregulated in ExoIL2-TAM-exosomes-	Inhibited cancer progression	(16)
miR-92a-2-5p	TAM	Reduced AR expression and regulated PHLPP/p-AKT/β-catenin signaling pathway	Enhanced cancer progression	(122)
miR-23a-3p	TAM	Increased VEGF and IL-4, which in turn led to further recruitment of M2 macrophages	Enhanced tumor angiogenesis	(124)
miR-200c-3p	TAM	Activated PI3K/AKT signaling pathway	Promoted Sorafenib resistance	(127)
circTMEM181	Tumor cell	Sponged miR-488-3p and promoted adenosine pathway.	Promoted T cell exhaustion and resistance to anti-PD-1 therapy	(131)
miR-1246	Tumor cell	miR-1246 was transferred by exosomes	Enhanced M2 macrophage polarization and inhibited the function of T cells	(132)
miR-146a-5p	Tumor cell	miR-146a-5p was induced by SALL4 and activated NF-κB pathway	Enhanced M2 macrophage polarization and inhibited the function of T cells	(133)
miR-23a-3p	Tumor cell	ER stress facilitated the release of exosomal miR-23a-3p and enhanced the expression of PD-L1 by regulating PTEN/PI3K signaling pathway	Inhibited T-cell function	(134)

MiRNAs are a highly conserved class of tissue-specific, small ncRNAs that play a crucial role in maintaining cellular homeostasis through negative regulation of gene expression. RNA sequencing analysis has revealed that miRNAs represent the predominant components in microvesicle obtained from human plasma. Exosomal miR-21-5p derived from tumor cells is associated with macrophage polarization and poor prognosis of HCC patients (89). Exosomal miR-21-5p inhibited Ras homolog family member B (RhoB) production and suppressed MAPK pathway, ultimately leading to M2-like macrophage polarization and HCC progression (89). Moreover, exosomal miR-21-5p also modulated specific protein 1 (SP1)/X-box binding protein 1 (XBP1), thus enhancing the polarization states of M2 macrophages and affecting the progression of HCC (90). Moreover, exosomal miR-452-5p directly targeted tissue inhibitors of metalloproteinases 3 (TIMP3) to induce M2 phenotype TAMs proliferation and polarization, representing a promising miR-452-5p/TIMP3 axis in HCC therapy (91). Additionally, exosomal miR-4669 contributed to the polarization of M2 macrophages by increasing sirtuin 1, which led to acquired resistance to sorafenib, promoted tumor aggressiveness and immunosuppressive tumor microenvironment, thus influencing the recurrence of HCC (92). In line with this, Xu et al. reported that miR-200b-3p exosomes downregulated zinc finger E-box binding homeobox 1 (ZEB1) expression and promoted IL-4 production, which trained macrophage polarization into M2-like phenotype and activated JAK/STAT pathway (93). ZEB1 functions as an essential transcriptional factor that is implicated in the epithelial-mesenchymal transition (EMT) (94). M2-like TAMs significantly upregulated the proviral Integration site for Moloney murine leukemia virus 1 (PIM1) and VEGFα expression, resulting in the activation of MEK/ERK signaling pathway and augmented cell EMT and metastasis in the setting of HCC (93). In line with this, exosomes enriched with circUPF2 from HCC cells facilitated the formation of the IGF2BP2-SLC7A11 ternary complex, which stabilized SLC7A11 mRNA, leading to increased sorafenib resistance and inhibited ferroptosis (95). Therefore, targeting exosomal circUPF2 may present a novel strategy for treating HCC.

Long non-coding RNAs (lncRNAs) are a class of ncRNAs that exceed 200 nucleotides in length and possess diverse functions both in the nucleus and the cytoplasm. Exosomal lncRNAs have been identified as signaling mediators that coordinate cellular functions. Li et al. reported that exosomal lncRNA TUC339 contributed to M1/M2 polarization by regulating cytokine-cytokine receptor binding (96). LncRNA HMMR-AS1 was notably induced by hypoxia-inducible factor-1 alpha (HIF-1α) and was associated with poor prognosis (97). Exosomes that carried HMMR-AS1 facilitated the M2 polarization of macrophages through sponging miR-147a and abrogating the degradation of ARID3A, thereby promoting HCC cell proliferation and growth (97). Furthermore, HCC cells secreted exosomal lncRNA HEIH that triggered macrophage polarization by targeting the miR-98-5p/STAT3 axis, which might shed light on the HCC treatment (98). Highly expressed lncRNA FAL1 in serum exosomes were observed in HCC patients and could promote tumor progression *in vivo*(99). It significantly induced M2 polarization of macrophages and subsequently activated the Wnt/β-catenin pathway, thus holding immense potential for novel strategies against HCC (99).

Metabolic reprogramming is a defining characteristic of cancer cells, promoting their growth and survival (100). LncRNA SLC16A1-AS1, derived from HCC exosomes, promoted the malignant progression of HCC by modulating macrophage polarization toward the M2 phenotype. Mechanistically, SLC16A1-AS1 enhanced the stability of SLC16A1 mRNA in macrophages (101). As a lactate transporter, SLC16A1 facilitated lactate influx, activating the c-Raf/ERK signaling pathway, which driven M2 polarization. In turn, M2 macrophages secreted IL-6, which activated the STAT3 pathway in HCC cells, inducing METTL3 transcription. This process increased m6A methylation and stability of SLC16A1-AS1. The reciprocal signaling between SLC16A1-AS1 and IL-6 in HCC cells and M2 macrophages promoted the proliferation, invasion, and glycolysis of HCC cells (101). Additionally, miR4458HG activated the glycolytic pathway, and promoted the polarization of TAMs in experimental models. Mechanistically, miR4458HG interacted with IGF2BP2, a key m6A RNA reader, enhancing the stability of target mRNAs such as HK2 and GLUT1, thereby impacting HCC glycolysis. Additionally, miR4458HG derived from HCC could be encapsulated in exosomes, further promoting the polarization of TAMs by increasing ARG1 expression (102). Similarly, Ji and colleagues revealed that lncRNA ZFPM2-AS1 was enriched in tumor cell-derived exosomes, which augmented the abilities and stemness of HCC cells by contributing to macrophage polarization (103). Further mechanistic studies have demonstrated that exosomal ZFPM2-AS1 regulated glycolysis by targeting the miRNA-18b-5p/PKM axis in a manner dependent on HIF-1α (103). These findings emphasized that exosomes serve as a signaling molecule that regulated metabolic regulation and macrophage polarization, suggesting that exosome could be a viable target for therapeutic intervention in HCC.

Exosomal proteins play an essential role in tumor development and progression. The expression of human AlkB homolog H5 (ALKBH5) was found to be enriched in liver cancer stem cells (LCSCs), potentially enhancing tumor growth and metastasis. Mechanistic studies have demonstrated that ALKBH5 significantly upregulated the expression of SPY-related high mobility group box 4 (SOX4) by inhibiting its N^6^-methyladenosine (m6A) modification, which in turn facilitated the transcriptional activation of sonic hedgehog (SHH) expression, thereby stimulating the SHH signaling pathway (104). Additionally, the exosomal ALKBH5 secreted by CD133^+^ HCC cells enhanced macrophage M2 polarization by promoting CCL5 secretion, upregulating IL-8 and mediating the upregulation of CPT1A (105–107). But in a recurrent spontaneous abortion model, overexpressed ALKBH5 reduced stromal VEGF secretion and impaired M2 macrophage differentiation and recruitment (108), thus the molecular mechanisms underlying macrophage polarization mediated by ALKBH5 awaited further investigation. Liu et al. examined the relationship between FTCD expression and immune cell infiltration using The Cancer Genome Atlas Program (TCGA) dataset and discovered that FTCD demonstrated a significant positive correlation with macrophage infiltration (109). Moreover, FTCD was considered a key potential exosome-related biomarker by stimulated macrophages exhibiting polarization towards the M1 type, leading to inhibited HCC growth (109). Knockdown of exosomal proteasome subunit alpha 5 (PSMA5) derived from HCC cells impeded M2 macrophage polarization via abrogating JAK2/STAT3 signaling pathway, leading to inhibited tumor cell proliferation, invasion, and migration (110).

## 5 TAMs-derived exosomes impact cellular functions in HCC

**Figure 4:**
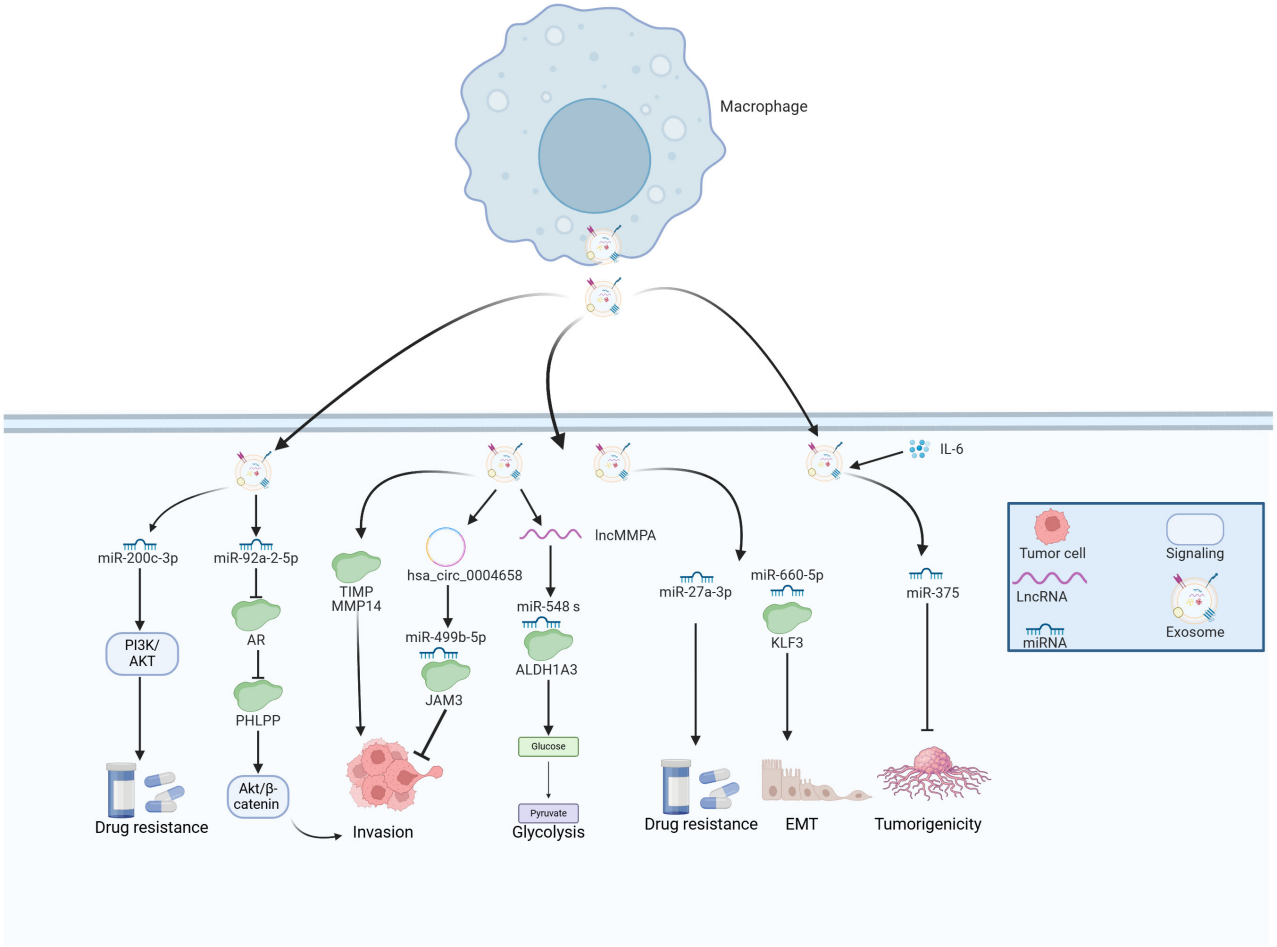
The emerging role of TAM-derived exosomes in HCC progression. TAMs secret various exosomes that contain multiple molecules, which play essential roles in the tumor growth, metastasis, glycolysis and drug resistance of HCC.

Exosomes derived from macrophages have demonstrated promise in targeting HCC cells. Increasing evidence suggests that these TAM-derived exosomes play a critical role in regulating cell proliferation, invasion, metastasis, metabolic reprogramming, and immune response (**Figure 4**).

LncMMPA was a myeloid-derived lncRNA that has been identified as a regulator for M2 macrophage polarization based on the scRNA-seq method (17). Further investigations have reported that the majority of extracellular lncMMPA existed within exosomes and the transfer of exosomal lncMMPA might take place between TAMs and Hep3B cells. The validation experiments indicated that exosomal lncMMPA significantly promoted the glycolytic pathway and cell proliferation by sponging miR-548 s and upregulating ALDH1A3 expression (17). The recombination signal binding protein-Jκ (RBPJ) functioned as a transcriptional regulator in the Notch signaling pathway, which has been implicated in various subsets of TAMs in HCC (111). Zhang et al. examined RBPJ overexpression in macrophages and its effects on HCC cells. Using circRNA microarray analysis, exosomal hsa_circ_0004658 was the most differentially regulated exosomal ncRNA in RBPJ overexpressed TAMs. Exosomal hsa_circ_0004658 demonstrated the ability to inhibit proliferation and migration while promoting apoptosis in HCC cells by targeting miR-499b-5p/JAM3 axis (112), which could function as a promising biomarker and therapeutic target for treating HCC. MiR-27a-3p was found to be upregulated while thioredoxin-interacting protein (TXNIP) was downregulated in HCC cells (113). Moreover, exosomes secreted by M2 macrophages were shown to further increase the levels of miR-27a-3p, which significantly enhanced tumorigenicity, stemness, and drug resistance of HCC cells (113).

Myeloid cell nuclear differentiation antigen (MNDA) belongs to the family of hematopoietic interferon-inducible nuclear proteins characterized by a pyrin domain (114). This protein is capable of regulating programmed cell death and inducing inflammatory responses (115, 116). MNDA acted as an independent prognostic factor and was predominantly expressed in M2-like TAMs, where it enhanced their polarization. Furthermore, MNDA-stimulated M2 TAMs secreted multiple exosomal proteins including MMP14, and TIMP, which promoted cell invasion, migration, and metastasis in HCC models (117). Tian et al. sought to explore the effects of miR-660-5p-modified M2-derived exosomes on the progression of HCC by regulating Kruppel-like factor 3 (KLF3). They observed elevated levels of miR-660-5p and decreased levels of KLF3 in HCC tissues, where increased levels of exosomal miR-660-5p facilitated the growth and EMT of HCC cells, an effect that could be reversed by overexpressing KLF3 (118). Furthermore, miR-660-5p-loaded M2 TAM exosomes bolstered the tumor-forming capacity in HCC mouse models, indicating that exosomal miR-660-5p from M2 TAMs significantly contributed to HCC tumorigenesis via modulating KLF3 (118). Moreover, miR-6876-5p within CD63-high macrophage was recognized as a key mediator, promoting EMT by targeting PTEN and activating the AKT signaling pathway. Additionally, exosomal miR-6876-5p accelerated tumor growth and metastasis in the setting of HCC (119). Chen and colleagues isolated TAM-derived exosomes from HCC tissues, and their exosomes were either treated with IL-2 (ExoIL2-TAM) or left untreated (ExoTAM). Among them, miR-375 was found to be upregulated in ExoIL2-TAM-exosomes and markedly decreased HCC cell tumorigenicity. These findings shed light on the mechanisms through which IL-2 inhibits HCC progression and underscores the potential clinical significance of exosomal miR-375 released by TAMs (16).

Androgen (AR) signaling plays a crucial role in the initiation and progression of HCC (120). Hypoxia could induce the phenotype of cancer stem cells by regulating the Androgen receptor (AR)-miR-520f-3p-SOX9 cascade, which resulted in acquired resistance to sorafenib (121). However, the relationship between AR and the TAMs during HCC development remains ambiguous. One recent study performed by Liu et al. reported that TAMs modified the expression of miR-92a-2-5p in exosomes, which reduced AR expression and subsequently regulated the pleckstrin homology domain leucine-rich repeat protein phosphatases (PHLPP)/p-AKT/β-catenin signaling pathway, leading to enhanced the invasive capabilities of HCC cells in preclinical models (122).

Cancer cells depend on oxygen and nutrients for survival and proliferation, necessitating their proximity to blood vessels to gain access to the circulatory system, which termed angiogenesis that could promote tumor progression (123). Exosomes derived from M2 macrophages were taken up by both HCC cells, enhancing vascular permeability, and promoting angiogenesis. Importantly, levels of miR-23a-3p were significantly elevated in M2-derived exosomes, with hnRNPA1 playing a key role in the packaging of miR-23a-3p into these exosomes. Moreover, HCC cells co-cultured with M2-derived exosomes released increased amounts of VEGF and IL-4, which in turn led to further recruitment of M2 macrophages and enhanced tumor angiogenesis (124).

Sorafenib serves as a first-generation multi-targeted tyrosine kinase inhibitor, demonstrated remarkable antiangiogenic and antiproliferative effects on tumor cells, leading to extended survival rates in advanced HCC patients (125). Treatment with sorafenib led to a reduction of tumor vessels formation and the exhaustion of pericytes, which may promote the recruitment of TAMs (126). In HCC patients, a positive correlation was observed between M2 macrophage scores and sorafenib efficiencies. Moreover, exosomes from M2 macrophages containing miR-200c-3p were found to promote acquired resistance to sorafenib by activating the PI3K/AKT signaling pathway (127). The study offers valuable insights into the role of M2 macrophages and their exosomes in sorafenib resistance and underscores the therapeutic potential of targeting this molecular pathway.

Recent studies have revealed the association between GPI anchored proteins and exosomes. Adiponectin bound to T-cadherin, a unique GPI-anchored cadherin on MSCs, promoting exosome biogenesis and secretion. Furthermore, increasing plasma adiponectin levels through pharmacological or adenovirus-mediated genetic approaches significantly enhanced the therapeutic effects of MSCs (128). These findings highlight the critical role of adiponectin in mesenchymal progenitor cell-mediated organ protection. Direct studies on the relationship between GPI-anchored proteins and TAM-derived exosomes remain scarce. However, given their essential roles in signal transduction and immune regulation, this area offers significant potential for future research and practical applications.

## 6 Crosstalk between exosomes and TAMs in HCC immune microenvironment

Immunotherapy, particularly through immune checkpoint inhibitors (ICIs), constitutes a major advancement in the development of oncology therapeutics over the past few years, which has the potential to provide significant advantages in clinical management of HCC (129). However, therapeutic resistance to ICIs including antibodies blocking programmed cell death 1 protein (PD-1)/programmed cell death 1 ligand 1 (PD-L1) pathways are emerging, leading to treatment failure and progressive disease in HCC patients (130). Mechanistic studies have revealed that immunosuppressive TME with exhaustion of T cells has been recognized as a critical factor that contributes to immunotherapy resistance in HCC. Exhausted T cell phenotype partially stems from excessive accumulation of TAMs, and the activated adenosine signaling with upregulated expression of CD73 and CD39, in which exosomes might play an essential role in immune regulation. High levels of exosomal circTMEM181 sponged miR-488-3p and upregulated CD39 expression in macrophages, synergistically promoting the activation of the adenosine pathway by cooperating with CD73 expression, thereby leading to T cell exhaustion and resistance to anti-PD-1 therapy in HCC patients (131).

Moreover, exosomes play a significant role in modulating tumor progression by educating immune cells within the microenvironment. Specifically, miR-1246 was transferred to macrophages through exosomes, guiding them toward a tumor-supporting phenotype and consequently establishing the immunosuppressive TME (132). Exosomal miR-146a-5p originated from HCC cells significantly promoted M2 macrophage polarization by activating the NF-κB pathway and subsequently inducing inflammatory factors, which induced immunosuppressive microenvironment by upregulating the expression of inhibitory receptors in T cells (133). Further investigations demonstrated that Sal-like protein-4 (SALL4) mediated the transcriptional activation of miR-146a-5p, and promoted its cellular delivery via exosomes. Blocking the SALL4/miR-146a-5p axis reversed the T cell exhaustion, which provided a promising therapeutic target for HCC patients (133).

**Figure 5:**
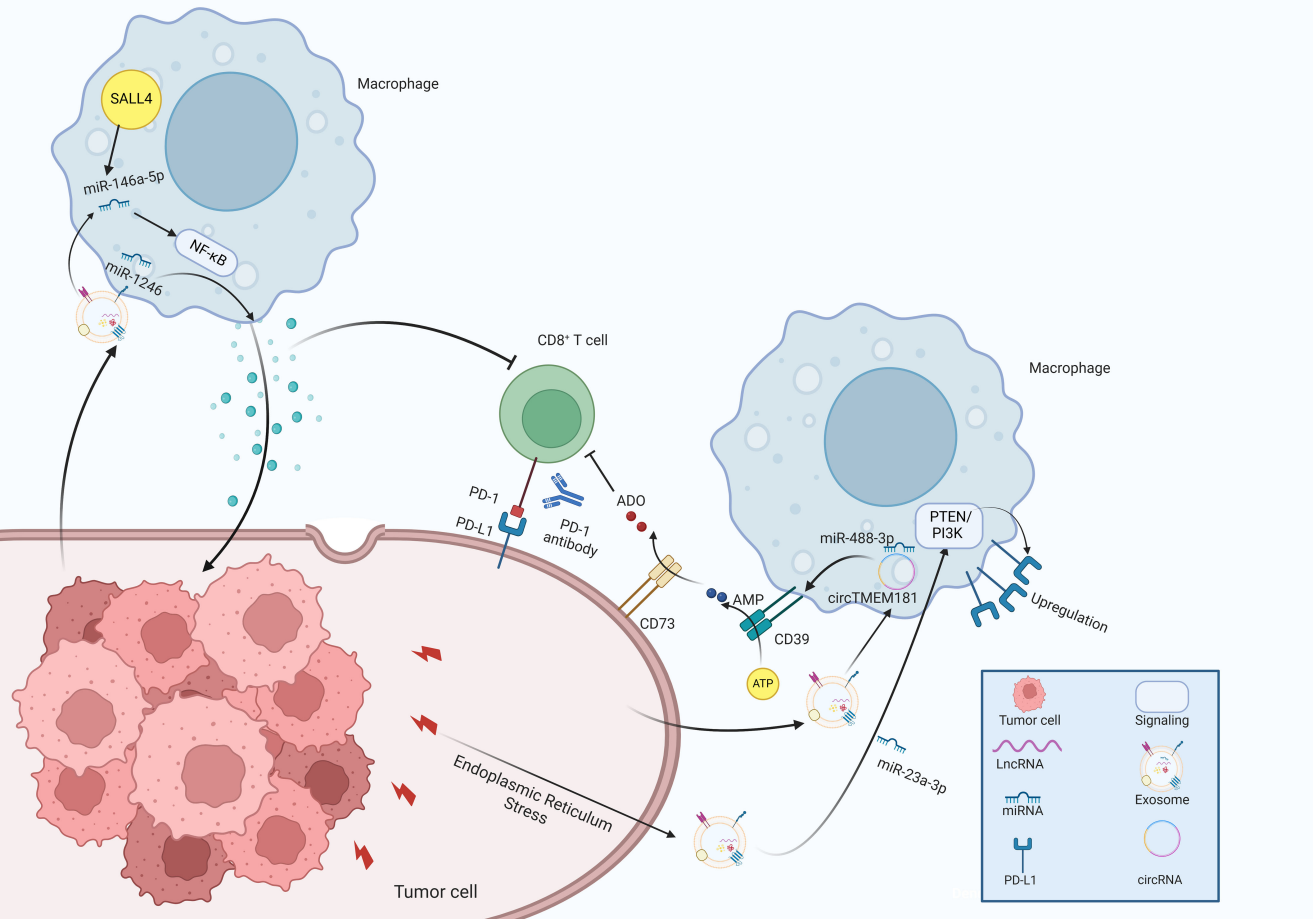
The molecular mechanisms of exosomes in immune regulation within the tumor microenvironment of HCC.

Endoplasmic reticulum (ER) stress plays a crucial role in preserving cell survival. Moreover, the activation of ER stress in immune cells is thought to influence the functionality of infiltrating immune cells, subsequently facilitating tumor growth. For example, Liu et al. reported that ER stress facilitated the release of exosomal miR-23a-3p and enhanced the expression of PD-L1 in TAMs by regulating the PTEN/PI3K signaling pathway, which subsequently inhibited T-cell function (134). Additionally, glycosylphosphatidylinositol (GPI) is a complex glycolipid broadly expressed across eukaryotic species (128). These findings have elucidated the essential of the crosstalk between exosomes and TAMs in the modulation of HCC TME (**Figure 5**).

## 7 Emerging role of exosomes in cancer therapy

Exosomes derived from macrophages possess the capability to transfer cargo to recipient cells, positioning them as promising candidates for targeted drug delivery and nanomaterial transport. These exosomes demonstrate outstanding biocompatibility, which enhance the ability of drugs to traverse natural barriers. The significant example was the engineering of exosomes to produce a fusion protein comprising the iRGD peptide (CRGDKGPDC), which targeted the αγ integrin, along with LAMP-2B. These engineered exosomes were capable of selectively delivering KRAS siRNA to non-small cell lung cancer cells that expressed the αvβ3 integrin, effectively downregulating the KRAS gene, inhibiting tumor proliferation, and demonstrating negligible toxicity. Sonodynamic therapy (SDT) presents a potential approach for tumor ablation through the activation of sonosensitizers in conjunction with ultrasound irradiation, making it promising for glioblastoma (GBM) therapy. Wu et al. developed a biodegradable nanoplatform (CSI), encapsulating catalase in silica nanoparticles (NPs) (135). They subsequently modified CSI with AS1411 aptamer-coated macrophage exosomes (CSI@Ex-A), which remarkably enhanced blood-brain barrier penetration and promoted specifically targeting tumor cells. Tumor cell endocytosis triggered the biodegradation of CSI@Ex-A, alleviating hypoxic TME and boosting SDT effectiveness with long circulation time, presenting a promising nanoplatform for clinical application (135). Yan et al. isolated exosome-like nanovesicles from B. javanica (designated as BF-Exos) and examined their effects and underlying molecular mechanisms in triple-negative breast cancer (TNBC). BF-Exos successfully transferred ten functional miRNAs to tumor cells, significantly hindering both the growth and metastasis of these cells by modulating the PI3K/Akt/mTOR signaling pathway and promoting ROS/caspase-mediated apoptosis (136). Moreover, cancer stem cells and MSCs-derived exosomes influenced signaling pathways associated with tumor progression *in vivo*, suggesting that they can sever as potential targets in HCC therapy (137). Xu et al. encapsulated doxorubicin (Dox) within Exos derived from human placental and modified these Exos with carboxylated Fe^3^O^4^ NPs to develop an Exo-Dox-NP delivery system. As a drug delivery vehicle, Exo-Dox-NPs significantly enhanced Dox uptake by tumor cells, exhibiting strong targeting specificity. Furthermore, Exo-Dox-NPs effectively inhibited the migration of cancer cells, with this formulation showing the highest anti-tumor activity (138). Yim et al. introduced a new tool for intracellular delivery of target proteins termed EXPLORs. By incorporating a blue light-controlled reversible protein-protein interaction module into the natural process of exosome biogenesis, they could effectively load cargo proteins into newly formed exosomes. Treatment with protein-loaded EXPLORs significantly enhanced the intracellular levels and functional capacity of these proteins in recipient cells, both *in vitro* and *in vivo*, which underscored the potential of EXPLORs as an effective mechanism for the intracellular transfer of protein-based therapeutics into target cells and tissues (139). Furthermore, a lipid-like prodrug of docetaxel (DSTG) featuring a reactive oxygen species (ROS)-cleavable linker, along with a lipid-conjugated photosensitizer (PPLA), spontaneously co-assemble into nanoparticles that acted as the lipid cores of the hybrid exosomes (HEMPs and NEMPs). These nanoparticles were subsequently encapsulated within membranes derived from adipocytes, enhancing their affinity for HCC cancer cells. Experimental studies demonstrated that HEMPs not only improved the bioactivity of the prodrug and prolonged its circulation time in the bloodstream but also effectively inhibited tumor growth by selectively targeting cancer cells. The self-facilitated synergistic drug release further enhanced the antitumor efficacy, leading to significant tumor growth inhibition with minimal side effects, suggesting a promising avenue for the development of targeted therapeutics for HCC (140).

Monoclonal antibodies (mAbs) that target specific molecules can be incorporated onto the surface of exosomes, functioning as potent “tools” to stimulate antitumor immune responses. For example, Nie et al. synthesized nano-bioconjugates by utilizing pH-sensitive linkers to conjugate Azide-modified M1 macrophage-derived exosomes with dibenzocyclooctyne-modified antibodies targeting CD47 and SIRPα, which regulated the “don’t eat me” pathway in macrophages. In the acidic TME, the linkers underwent cleavage, resulting in the release of specific antibodies, which significantly enhanced macrophage phagocytosis (47). Recently, a novel type of engineered exosome, inspired by chimeric antigen receptor macrophage cells (CAR-M), has garnered attention due to its superior antitumor efficacy and reduced incidence of adverse events. Jiang et al. utilized exosomes derived from CAR-M cells as the targeted drug carrier, which were enriched with a high concentration of CXCL10. Subsequently, CAR-exosomes were covalently loaded with the chemotherapeutic agent SN-38, establishing a novel antibody-drug conjugates (ADCs), which markedly promoted the immunological activation and enhanced the recruitments of TAMs, outperforming traditional ADCs in antitumor effects, providing novel insights into future drug development (141). The above findings highlight the essential role of exosomes in cancer therapy in the preclinical settings. Additionally, more investigations are focused on the clinical utilization of exosomes in cancer therapy. For example, an ongoing clinical trial (NCT05575622) performs the detection of exosomal PD-L1 and LAG-3 proteins. The goal is to characterize the functional marker profiles associated with immunotherapy in the peripheral blood of HCC patients and to provide a comprehensive assessment of their responsiveness to such treatments. Another clinical trial (NCT06342414) aims to develop and validate a liquid biopsy that assesses circulating exosomal miRNAs for indirect sampling of tumor tissue present in the bloodstream, aiming to create a cost-effective, non-invasive assay suitable for clinical application, enhancing the sensitivity and specificity for diagnosing HCC. Moreover, camel milk contains various exosomes that hold immense potential for anti-cancer treatment (55, 142). Camel milk-derived exosomes exhibited a stronger anti-cancer effect on HCC cells by the induction of apoptosis and the suppression of inflammation and angiogenesis (143). Thus, these exosomes could act as safe adjuvants or carriers for the delivery of chemotherapeutics, enhancing their anti-cancer effects on HCC cells.

In addition, exosomes derived from macrophages may play an important role in reversing tumor resistance (**Table 2**). For example, exosomes from M1 macrophages loaded with cisplatin have been shown to enhance anticancer efficacy, specifically by inhibiting cancer cell growth, and increasing drug sensitivity (144). The expression of exosomal miR-301a-3p was elevated in the lenvatinib-resistant HCC cells, activating the PTEN/PI3K cascade in TAMs, which increased cell resistance to lenvatinib (145).

**Table 2:** The role of exosomes in the drug resistance of HCC.

Exosomal cargo	Donor cell	Mechanism	Effect	Reference
miR-301a-3p	Tumor cell	Activated the PTEN/PI3K/GSK3β/Nrf2 signaling pathway	Promoted lenvatinib resistance	(145)
circ 0008253	M2 macrophages	Promoted cell proliferation by regulating ABCG2 levels	Decrease oxaliplatin sensitivity	(146)
miR-222-3p	M2 macrophages	Downregulated TSC1 and activated the PI3K/AKT signaling pathway	Promoted chemoresistance	(147)
miR-4669	Tumor cell	Induced M2 macrophage polarization.	Promoted sorafenib resistance	(92)
miR-200c-3p	M2 macrophages	Activated PI3K/AKT pathway	Promoted sorafenib resistance	(127)

Moreover, exosomes isolated from M2 macrophages could transfer circ 0008253 to cancer cells, which possessed the ability to decrease oxaliplatin sensitivity and promote cell proliferation by regulating ABCG2 levels (146). Furthermore, Guo et al. performed a study that aimed to elucidate the downstream mechanisms by which exosomal miR-222-3p, delivered via exosomes derived from M2 macrophages, contributed to drug resistance (147). Both *in vivo* and *in vitro*, exosomal miR-222-3p from M2-polarized macrophages potentiated chemoresistance through the downregulation of TSC1 and the activation of the PI3K/AKT signaling pathway (147). In HCC, as we have discussed above, exosomal miR-4669, and miR-200c-3p also promoted sorafenib resistance, presenting promising targets for precision medicine (92, 127). In xenograft and liver metastasis models, the sequential administration of folic acid-modified milk exosomes loaded with c-kit siRNA (FA-mExo-siRNA-c-kit) followed by gefitinib resulted in decreased tumor growth and improved survival rates. Mechanistically, c-kit was identified as a regulator of the AKT/mTOR/4EBP1/eIF4E pathway, promoting both stemness and resistance to gefitinib in lung cancer cells. The utilization of FA-mExo-siRNA-c-kit might enhance patient outcomes by overcoming gefitinib resistance, warranting further investigation into this approach (148).

## 8 Challenges in the therapeutic application of exosomes

Despite considerable progress, various challenges continue to hinder the therapeutic application of exosomes. Firstly, exosomes are diverse and widely found entities; however, their complexities remain incompletely understood, especially regarding the mechanisms of cargo sorting into exosomes and the release of that cargo into cells after exosome internalization (149). While recent studies have largely concentrated on protein sorting, it appears that the primary functions of exosomes are more associated with RNA delivery (150). Thus, understanding the mechanisms behind RNA sorting hold considerable promises for the development of various applications.

In this field, there is currently no standardized protocol for exosome isolation. Ultracentrifugation remains the most common technique for separating exosomes; it is essential to recognize that while ultracentrifugation can effectively concentrate substances with similar density and size, it does not allow for precise differentiation of exosomes (151). Ultracentrifugation has several advantages, including established technology, compatibility with a wide range of samples, and low operational costs (152). However, it suffers from low reproducibility and the potential to damage the exosomes, rendering it inappropriate for clinical applications (153). Additionally, efficient isolation of tumor-derived exosomes can be achieved by targeting specific proteins found in these exosomes, such as EpCAM and anti-A33 (154). Currently, immunomagnetic beads are commonly used; these antibody-coated beads selectively capture the corresponding exosomes, enabling their differentiation from unbound impurities via magnetic separation (155). Moreover, microfluidics, capable of manipulating small fluid volumes (microliters), offers advantages such as rapid separation, high throughput, and minimal sample requirements, making it ideal for isolating exosomes from limited biological samples (156).

Other significant challenges involve the scalability of exosome production. Typically, exosomes are produced in limited quantities, and the processes for their isolation and purification can be time-consuming and costly (157). To enhance the clinical application of exosomes, it is essential to develop scalable production methods that can generate substantial amounts of exosomes in a cost-effective manner (158). Moreover, each stage of exosome biogenesis is mediated by various mechanisms that exhibit high variability, leading to the observed heterogeneity of exosomes (159). The heterogeneity of exosomes and the complexity of the *in vivo* environment limit their precise delivery and expected outcomes (160). The source and composition of exosomes can vary due to different cell types, disease states, and microenvironmental factors, making the strategy of using exosomes as drug delivery systems in cancer treatment relatively complex (161). The TME contains various cell types, signaling molecules, and intercellular interactions, all of which collectively influence tumor progression and treatment response. This complexity means that even if a treatment is effective in preclinical stages, the actual application to patients may lead to different therapeutic outcomes due to various individual differences. Therefore, when developing personalized treatment plans, it is essential to systematically consider these complex factors to determine the most suitable therapeutic targets and strategies. To tackle this problem, autologous exosomes obtained from cancer patients have surfaced as a promising delivery system, owing to their exceptional ability to specifically target cancer cells (162). Autologous plasma-derived exosomes are readily accessible and can circumvent the immune responses often elicited by exogenous exosomes (163). Ran et al. constructed a biological scaffold based on autologous plasma exosomes, which were loaded with neuron-targeting peptides and growth-promoting peptides (162). By integrating both efficacy and safety, the autologous plasma exosome-based personalized treatment has exhibited significant potential for biomedical applications, which aided in broadening the utilization of combinatory peptides and autologous exosomes derived from human plasma in the context of human disease treatment (162). Jiang et al. loaded gemcitabine into autologous exosomes to facilitate cellular uptake and enhance the cytotoxicity of gemcitabine, leading to significant inhibition of tumor growth and reduction of tumor recurrence in mice. This approach may offer important implications for personalized therapy in cancer (163).

Moreover, a comprehensive assessment of the safety of exosome-based therapies is necessary. The *in vivo* function and safety of exosomes continue to be a subject of controversy. Given their biological activity, it is important to assess the safety of exosomes when utilized as delivery vehicles. For example, exosomes derived from TAMs may carry components that facilitate cancer cell growth and metastasis, which can pose risks of enhancing tumorigenesis (112). Additionally, potential off-target effects and unintended consequences associated with exosome therapy warrant careful investigation (164). Standardized production processes ensure high purity and consistency of exosomes, thereby reducing the presence of potential contaminants and improving the potential toxicities (80). Moreover, conducting comprehensive characterization of exosomes, including their size, surface markers, and proteomic analysis, to ensure they meet therapeutic standards (165). Consequently, it is essential to conduct more preclinical evaluations on exosomes that encompass assessments of pharmacokinetics, and toxicity profiles to minimize any potential adverse effects, which can promote the translation into clinical course.

## 9 Conclusion

Based on these emerging studies, the prospects for HCC therapy based on exosomes hold immense potential for clinical application. Exosomes derived from TAMs or tumor cells are skilled mediators of immune response, and their relatively straightforward manipulation of TME provides notable advantages, laying the foundation for future therapeutic uses in HCC. While our current comprehension of the specific mechanisms and functions of exosomes is still limited, there is a progressive unveiling of the “mysterious veil” that envelops the TME of HCC. Moreover, there are several challenges for hampering the clinical application of exosomes so far. Developing efficient strategies for exosome isolation, as well as establishing the safety and efficacy of cancer therapy based on exosome, are crucial research areas that need further attention. Further research should explore innovative engineering approaches for exosomes, such as genetic or surface modifications, to enhance their targeting capabilities and therapeutic potential. Furthermore, exploring the potential of integrating exosomes with established chemotherapy or immunotherapy agents could enhance treatment efficacy and reduce side effects, especially in certain cancer contexts. Recent advancements in nanotechnology may be crucial in this regard, offering substantial benefits for clinical translation and holding considerable promise for HCC treatment.
